# Thriving or Withering? Plant Molecular Cytogenetics in the First Quarter of the 21st Century

**DOI:** 10.3390/ijms26147013

**Published:** 2025-07-21

**Authors:** Elzbieta Wolny, Luis A. J. Mur, Nobuko Ohmido, Zujun Yin, Kai Wang, Robert Hasterok

**Affiliations:** 1 Plant Cytogenetics and Molecular Biology Group, Faculty of Natural Sciences, Institute of Biology, Biotechnology and Environmental Protection, University of Silesia in Katowice, 40-032 Katowice, Poland; 2 Department of Life Sciences, Aberystwyth University, Edward Llwyd Building, Aberystwyth SY23 3DA, UK; lum@aber.ac.uk; 3 Graduate School of Human Development and Environment, Kobe University, Kobe 657-8501, Japan; ohmido@kobe-u.ac.jp; 4 State Key Laboratory of Cotton Bio-Breeding and Integrated Utilization, Institute of Cotton Research, Chinese Academy of Agricultural Sciences, Anyang 455000, China; yinzujun@caas.cn; 5 Western Agricultural Research Center, Chinese Academy of Agricultural Sciences, Changji 831100, China; 6 School of Life Sciences, Nantong University, Nantong 226019, China; kwang5@126.com

**Keywords:** chromosome, chromosome markers, cytogenetics, cytomolecular analysis, interphase nucleus, FISH, fluorescence in situ hybridisation, karyotype, plant molecular cytogenetics, plant nuclear genome

## Abstract

Nearly four decades have passed since fluorescence in situ hybridisation was first applied in plants to support molecular cytogenetic analyses across a wide range of species. Subsequent advances in DNA sequencing, bioinformatic analysis, and microscopy, together with the immunolocalisation of various nuclear components, have provided unprecedented insights into the cytomolecular organisation of the nuclear genome in both model and non-model plants, with crop species being perhaps the most significant. The ready availability of sequenced genomes is now facilitating the application of state-of-the-art cytomolecular techniques across diverse plant species. However, these same advances in genomics also pose a challenge to the future of plant molecular cytogenetics, as DNA sequence analysis is increasingly perceived as offering comparable insights into genome organisation. This perception persists despite the continued relevance of FISH-based approaches for the physical anchoring of genome assemblies to chromosomes. Furthermore, cytogenetic approaches cannot currently rival purely genomic methods in terms of throughput, standardisation, and automation. This review highlights the latest key topics in plant cytomolecular research, with particular emphasis on chromosome identification and karyotype evolution, chromatin and interphase nuclear organisation, chromosome structure, hybridisation and polyploidy, and cytogenetics-assisted crop improvement. In doing so, it underscores the distinctive contributions that cytogenetic techniques continue to offer in genomic research. Additionally, we critically assess future directions and emerging opportunities in the field, including those related to CRISPR/Cas-based live-cell imaging and chromosome engineering, as well as AI-assisted image analysis and karyotyping.

## 1. Introduction

**Figure 1:**
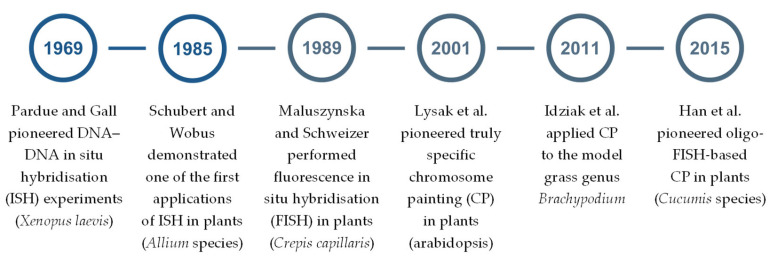
Milestones in the development and application of DNA–DNA in situ hybridisation, with a specific focus on plant research. The six milestones correspond to the following publications: [3,4,6,19,24,30]. Figure created using BioRender (https://BioRender.com, accessed on 15 July 2025).

Modern molecular cytogenetics integrates genetic, genomic, molecular, and cell biology approaches with advanced microscopic imaging techniques. Its flagship technique, DNA–DNA in situ hybridisation (ISH), enables the precise mapping of target sequences across a range of cytogenetic substrates, including mitotic and meiotic chromosomes at various stages of division, interphase nuclei, and extended chromatin fibres. Each of these substrates offers distinct advantages in terms of mapping resolution, individual chromosome identification, and suitability for addressing specific research questions [1,2]. ISH was first developed in the late 1960s for *Xenopus laevis* [3] (Figure 1) and was later applied to plants in the mid-1980s. However, its full potential was initially constrained by the technical limitations of non-fluorescent detection systems, such as autoradiographic [4] (Figure 1) and enzymatic [5]. In 1989, Maluszynska and Schweizer demonstrated the chromosomal localisation of ribosomal RNA (rRNA) genes on B chromosomes of *Crepis capillaris*, using biotinylated 18S and 25S ribosomal DNA (rDNA) probes, detected through the biotin–avidin–fluorescein isothiocyanate (FITC) system [6]. This work represents one of the earliest published applications of DNA–DNA fluorescence in situ hybridisation (FISH) in plants (Figure 1). The first attempts at in situ localisation of single- or low-copy chromosome-specific sequences followed relatively soon thereafter [7,8,9]. Nevertheless, early applications of FISH in plants primarily focused on mapping repetitive DNA sequences, including evolutionarily conserved elements (e.g., rDNA and T_3_AG_3_ telomeric motifs), as well as species- and genus-specific sequences such as total genomic DNA (gDNA), selected tandem repeats, and transgenes. These efforts initially used single-colour (e.g., [10,11,12]), then dual-colour (e.g., [13,14,15]), and occasionally multi-colour (e.g., [16,17]) visualisation. The breakthrough came in 2000 with the sequencing of the first plant nuclear genome—of the model angiosperm *Arabidopsis thaliana* (arabidopsis) [18]. Building on this, Lysak et al. employed collections of ordered bacterial artificial chromosome (BAC) clones containing large inserts of arabidopsis gDNA to specifically paint chromosomes—first in this species [19] (Figure 1), and later in its relatives [20]. This approach significantly advanced plant genome analysis by enabling, for example, comparative studies of karyotype structure and evolution, albeit at individual chromosome segment resolution [21]. It also allowed the spatial positioning of chromosomes within the interphase nucleus to be determined [22]. However, effective chromosome painting (CP) using this method was limited to a handful of small-genome plant species, such as tomato, potato [23], and representatives of the model grass genus *Brachypodium* [24,25] (Figure 1). This problem reflected the limited availability of sequenced plant genomes at the time, the technical complexity of BAC library construction and handling [26], and the difficulty in obtaining contiguous, chromosome-specific signals in plants with large and/or repeat-rich genomes. Many of these limitations were overcome with the advent and widespread application of probes based on single- or low-copy oligonucleotides in the so-called oligo-FISH approach, a technique originally developed for animal genomes (e.g., [27,28,29]) and later adapted for use in plants [30] (Figure 1). This approach relied on the development of bioinformatic tools for genome mining to identify suitable probe targets (for recent reviews, see [31,32]), advances in the commercial large-scale synthesis of customised oligonucleotides, and, most importantly, the rapid increase in the number of sequenced plant genomes [33,34]. These advances not only paved the way for CP in species that were previously considered challenging or even intractable (e.g., [35,36,37]), but also facilitated the relatively straightforward generation of FISH probes from virtually any sequence of interest.

FISH analyses can be supplemented by, or combined with, immunofluorescent approaches targeting chromatin modifications (for a comprehensive review, see [38]) or other nuclear components, thereby offering deeper insights into the developmental [39,40] and functional dynamics of the nucleus [41,42,43], as well as its epigenetic responses to experimentally induced stresses [44,45,46,47]. These techniques, supported by state-of-the-art microscopy platforms (for reviews, see, e.g., [48,49,50]), along with advanced image processing and analysis platforms (e.g., [51,52]), have yielded unprecedented insights into cytomolecular organisation of nuclear genomes across a wide spectrum of model, non-model, and crop plants. Notably, in a broader genomic context, FISH-based approaches using chromosome- or genome-specific probes have played a vital role in physically anchoring genome sequence assemblies to chromosomes, as demonstrated in foundational work [18] and subsequent earlier studies (e.g., [53,54]), and continue to do so in the most recent work (e.g., [55]).

**Figure 2:**
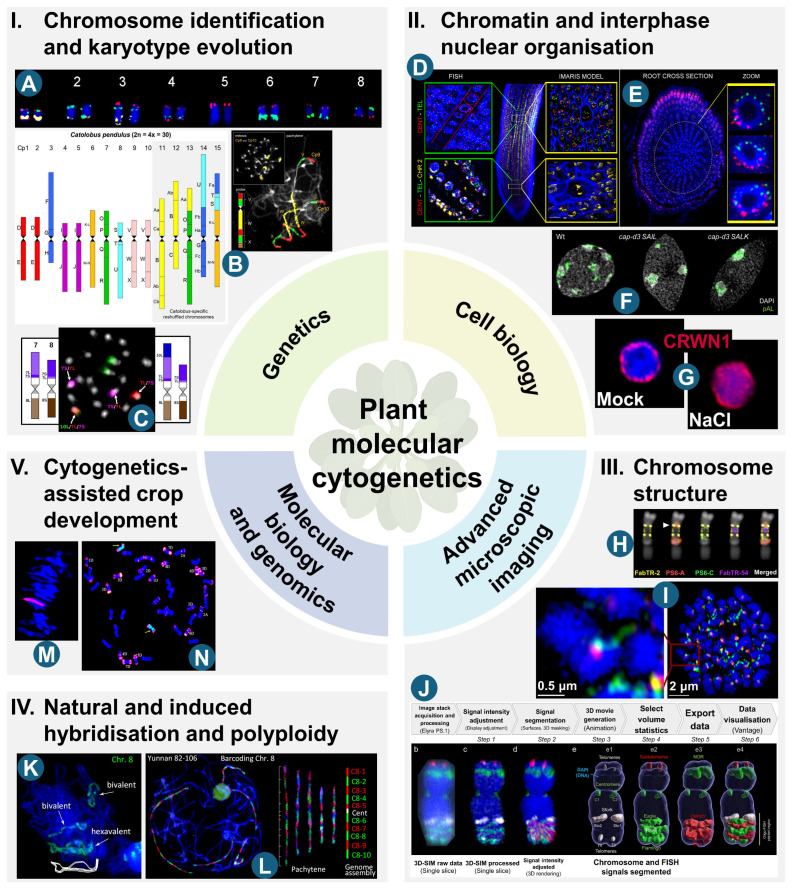
Current major areas in plant cytomolecular research (**I**–**V**). (**A**) Karyogram of *Cicer arietinum* CDC Frontiers (kabuli type) chromosomes, discriminated using FISH on mitotic metaphase chromosomes with a combination of painting probes: CAF-OP1 (green), CAF-OP2 (red), and 5S rDNA (yellow) (adapted from [58]). (**B**) Ideogram showing the chromosome-level structure of the *Catalobus pendulus* genome, based on CCP analysis, indicating the positions of 44 genomic blocks on its 15 chromosomes (Cp1–Cp15). The photomicrograph shows an example of CCP with mitotic and pachytene chromosomes Cp9 and Cp10 painted using arabidopsis BAC contigs representing ancestral genomic blocks V, W, and X, respectively (adapted from [59]). (**C**) Exemplary chromosome translocations identified by oligo-FISH on mitotic metaphase chromosomes of *Musa acuminata* ITC0660 ‘Khae (Phrae)’, using probes specific for the long arm of chromosome 10 and the short and long arms of chromosome 7 (adapted from [60]). (**D**,**E**) FISH with centromeric, telomeric, and chromosome 2-specific probes applied to ultra-thin root sections of *Oryza sativa*, prepared using a cryomicrotome (adapted from [61]). The images show evidence of Rabl configuration in xylem (**D**) and cortex (**E**) cells. (**F**) 3D-SIM maximum intensity projections of FISH with the pAL centromeric repeat on structurally preserved, acrylamide-embedded 4C leaf nuclei of arabidopsis WT and *cap-d3* mutants. The *cap-d3* mutation affects centromere association but does not alter their overall spatial arrangement within the nuclei (adapted from [62]). (**G**) Changes in the localisation of the arabidopsis nuclear lamina protein CRWN1 under salt stress (adapted from [63]). (**H**) Multicolour FISH labelling of the *Lathyrus sativus* homoeologue of pea chromosome 6 using PS6 painting probes, along with probes for the satellite repeats FabTR-54, which fills the gap in the PS6-C signal, and FabTR-2, which is associated with CENH3 chromatin in *L. sativus* (adapted from [64]). (**I**) Interaction of the centromeric protein CENH3 (red) with α-tubulin (green) in metaphase chromosomes of *Prionium serratum*, visualised using spatial SIM (adapted from [65]). (**J**) Overview of the image analysis workflow for examining the ultrastructure of the *Hordeum vulgare* 5H metaphase chromosome after FISH using centromeric, 35S rDNA-targeting, telomeric, subtelomeric, and 5HL-specific oligo probes (adapted from [66]). (**K**,**L**) Meiotic chromosome behaviour in the autodecaploid *Saccharum spontaneum* clone Yunnan 82–106 revealed using CP oligo probes (adapted from [67]). (**K**) Pairing configurations at pachytene showing ten copies of chromosome 8 forming two bivalents and one hexavalent. (**L**) Five bivalents of chromosome 8 with their detailed structure visualised using dual-colour barcoded painting FISH. (**M**) GISH using gDNA of *Agropyron cristatum* (red) reveals the presence and stable meiotic behaviour of the translocated *A. cristatum* 1P chromosome segment in the wheat background of the T1AS.1PL translocation line (adapted from [68]). (**N**) Visualisation of *Aegilops geniculata* chromosomes (indicated by arrows) at mitotic metaphase in a *Triticum aestivum–A. geniculata* substitution line, using GISH with *A. geniculata* genomic DNA (green) and FISH with the pTa535 D-genome-specific probe (red) (adapted from [69]). Figure created using BioRender (https://BioRender.com, accessed on 15 July 2025). All materials presented in this figure, except for the photomicrograph in panel (**M**), were published under the terms of the Creative Commons Attribution (CC-BY 4.0) licence (https://creativecommons.org/licenses/by/4.0/). The image in panel (**M**) has been published under an exclusive licence to Springer-Verlag GmbH Germany, with the authors of the original publication retaining copyright.

Meanwhile, rapid advances in sequencing technologies and bioinformatics have made large-scale genomic analyses faster and more affordable than ever before. According to published data [33,34], by the end of 2024, the total number of plant species with sequenced genomes had exceeded 1800, including 370 species sequenced and published for the first time that year. This milestone demonstrated the feasibility of the Ten Thousand Plant Genome Project’s objectives, which had been set only a few years earlier [56]. While these developments create opportunities to apply advanced cytomolecular approaches to a wide range of plant species, they also raise critical questions about the future of plant molecular cytogenetics, a field where techniques are often inherently low-throughput, difficult to standardise, labour-intensive, time-consuming, and largely resistant to automation (e.g., [2,57]). In this review, we summarise recent developments (2020–2025) in major areas of plant molecular cytogenetics, with particular focus on various aspects of chromosome identification and karyotype evolution, chromatin and interphase nuclear organisation, chromosome structure, hybridisation and polyploidy, and cytogenetics-assisted crop development (Figure 2). We also explore future prospects, including CRISPR (clustered regularly interspaced short palindromic repeats)/Cas (CRISPR-associated protein)-based live cell imaging and CRISPR/Cas chromosome engineering, together with their applications and limitations in plant cytomolecular research, as well as artificial intelligence (AI)-assisted image analysis and karyotyping.

## 2. Chromosome Identification and Karyotype Evolution

The identification of individual chromosomes and their segments within a given species has been, and continues to be, central to most cytomolecular analyses, regardless of their specific objectives. Unsurprisingly, this focus is consistent across a wide range of species, as evidenced in many recent publications (Table 1). To achieve this, robust chromosome markers are essential. As comprehensively reviewed for *Brachypodium* [70] and *Saccharum* [71], various FISH probes can serve this purpose. However, their effectiveness depends not only on the sequences employed and the availability of well-developed genomic resources, but also on the specific organisation of the target genome, including its size and the proportion of single- or low-copy sequences relative to repetitive DNA content. The most advanced studies, particularly those involving comparative analyses of the structure and evolution of individual chromosomes and entire karyotypes within related species, require markers capable of specifically painting entire chromosomes or their substantial segments, or, at the very least, providing a chromosome-unique barcode. In recent years, such chromosomal landmarks have become the gold standard in plant karyotyping (Table 1; section I of Figure 2). This progress can be partly attributed to the use of long-established BAC libraries, which have enabled, for example, the reconstruction of karyotype organisation within previously understudied groups of the Brassicaceae [59,72] (Figure 2B), annual [73] and perennial [74]. *Brachypodium* polyploids, as well as various representatives of the Fabaceae [75,76,77] and *Passiflora* [78].

Most current studies routinely rely on the application of oligo-FISH probe libraries, which either complement existing BAC-based resources (e.g., [58,79,80,81,82]) or have been developed for a range of both monocot (e.g., [83,84,85,86,87,88,89,90,91]) and eudicot (e.g., [92,93,94,95,96,97,98,99]) species, for which effective CP or comparative chromosome painting (CCP) would otherwise be limited or unavailable. Some studies primarily focus on chromosome identification within individual species, such as *Tripidium arundinaceum* [100] and *Gossypium hirsutum* [96]. However, currently available marker systems enable precise tracking of the structure and evolution of individual chromosomes and entire karyotypes across various taxonomic levels. These range from intraspecific analyses within *Cicer arietinum* [58] (Figure 2A), to intrageneric comparisons in *Brachypodium* [73,74], *Phaseolus* [76,101], *Vigna* [81,82], *Lupinus* [80], *Passiflora* [78], *Avena* [86], *Rhynchospora* [87], *Musa* [60,89] (Figure 2C), *Citrus* [92], *Fragaria* [93], *Ipomoea* [94,99], *Cucumis* [97,98], *Glycyrrhiza* [95], and *Silene* [102]. They also extend to complex intergeneric comparisons involving evolutionarily more or less distant groups, such as the *Saccharum* complex [90], and the Thlaspideae [72], Phaseoleae [103], and Triticeae [83,84,88] tribes. These comparative analyses have greatly advanced our understanding of nuclear genome evolution at the chromosomal level across diverse plant groups. For example, they have uncovered extensive, and in some cases complete, chromosomal synteny across a range of divergence times, such as between *Tripidium arundinaceum* and *Zea mays* (maize; ~18 million years; MYs), *Tripidium arundinaceum* and sorghum (~9 MYs) [100], within the genera *Citrus* (~9 MYs) [92] and *Glycyrrhiza* (~3–12 MYs) [95], and between *Brachypodium hybridum* and its putative diploid progenitors, *B. distachyon* (*Brachypodium*) and *B. stacei* (~0.14–1.4 MYs) [73]. On the other hand, numerous studies across various genera highlight the prevalence of species- and genus-specific chromosome rearrangements (CRs), such as duplications, inversions, and translocations, which contribute to karyotype diversification and speciation [24,58,72,75,76,79,80,87,88,90,97,98,102,104]. Particularly noteworthy are recent analyses employing CP in large-genome grasses [83,88], including allopolyploids such as *Campeiostachys* (*Elymus*) *nutans* [84], which has a genome size exceeding 10 Gb [55]. Thus, the evolutionary histories of entire genomes and individual chromosomes in species that were previously highly challenging, or even intractable, to study can now be elucidated.

Given the current availability and versatility of oligo-based probes, it is not surprising that the use of other types of FISH probes, long employed in chromosome identification and karyotyping, is gradually declining. For instance, 5S and 35S rDNA-targeting sequences, which are among the methodologically simplest, are now typically used only as auxiliary probes. ([58,72,75,76,77,78,79,81,85,90,91,92,93,94,95,96,100,101,105,106]; see Table 1 for details). Although such sequences may still be useful for preliminary comparative studies and in supporting molecular phylogenetic analyses in plant species where more specific chromosome landmarks are not yet available, as illustrated in the genus *Onobrychis* [107]. Other simple probes, for example, those based on gDNA, which is essential for genomic in situ hybridisation (GISH), are effectively obsolete as an approach (e.g., [72,85]). However, gDNA can be useful in the study of allopolyploids and in tracking individual chromosomes or chromosome fragments within alien genomic backgrounds. These applications are particularly important for the rapid integration of cytogenetic approaches into plant breeding, as discussed further in this review.

**Table 1:** A selection of recent research articles on chromosome identification and karyotype evolution.

Research Object	Research Approach	Aims and Main Findings	References
Thlaspideae (Brassicaceae): *Alliaria petiolata* (2x, 6x), *Didymophysa fenestrata*, *Graellsia saxifragifolia*, *G. stylosa*, *Parlatoria cakiloidea*, *Peltaria turkmena*, *Peltariopsis grossheimii*, *P. planisiliqua*, *Pseudocamelina glaucophylla*, *P. szowitsii*, *Thlaspi arvensa*	Multiple FISH approaches: CCP with *Arabidopsis thaliana* BAC contigs; GISH with gDNA of *Pa. cakiloidea* and *A. petiolata*; oligo-FISH with probes localising satDNAs, BAC-FISH with clones targeting 5S and 35S rDNA	Reconstruction of chromosomal organisation in the ancestral genome and analysis of karyotype structure and evolution in 12 Thlaspideae representatives; detection of genus- and species-specific CRs (e.g., pericentric inversions); evidence for allohexaploid origin of *A. petiolata* (6x) from diploid *A. petiolata* and *Pa. cakiloidea*	[72]
*Catolobus pendulus*	CCP with *Arabidopsis thaliana* BAC contigs	Karyotype organisation of an understudied species; the hypotetraploid *C. pendulus* genome originated from a whole-genome duplication in a genome resembling the ACK, followed by chromosomal rediploidisation	[59]
*B. distachyon*, *B. stacei*, *B. hybridum*	BAC-FISH with chromosome-specific *B. distachyon* clones	Reconstruction of chromosome evolution between genome D and S in annual *Brachypodium* diploids and their derived allopolyploid; complete chromosomal synteny observed between *B. hybridum* and its progenitors	[73]
*Brachypodium boissieri*, *B. mexicanum*, *B. phoenicoides*, *B. retusum*, *B. rupestre*	BAC-FISH with chromosome-specific *B. distachyon* clones	Chromosome identification in all species; reconstruction of chromosome evolution among the A1.1, A1.2, A2, E1, E2, and G genome in perennial *Brachypodium* polyploids; demonstration of ‘orphan’ genomes in the model grass genus	[74]
*Phaseolus vulgaris*, *Vigna aconitifolia*, *V. unguiculata*	BAC-FISH with chromosome-specific *P. vulgaris* and *V. unguiculata* clones; FISH with 5S and 35S rDNA-targeting probes	Intergeneric analyses of karyotype organisation; detection of macrosynteny breaks between *Vigna* and *Phaseolus*; CRs (duplications, inversions, and translocations) contribute to karyotype divergence in *Vigna*	[75]
*Phaseolus leptostachyus*, *P. macvaughii*	BAC-FISH with chromosome-specific *Phaseolus vulgaris* clones; FISH with 5S and 35S rDNA-targeting probes	Interspecific analyses of karyotype organisation; CRs observed in *P. leptostachyus* are not shared with *P. macvaughii*; only one nested chromosome fusion (chromosomes 10 and 11), is common to both; pericentric inversions detected in chromosomes 3 and 4 exclusively in *P. macvaughii*	[76]
*Macroptilium atropurpureum*, *M. bracteatum*, *M. erythroloma*, *M. gracile*, *M. lathyroides*, *M. martii*	BAC-FISH with chromosome-specific *Phaseolus vulgaris* clones; FISH with 5S and 35S rDNA-targeting probes	Interspecific analyses of karyotype organisation; BAC markers show synteny on orthologous chromosomes, while karyotype differentiation primarily driven by the number and distribution of rDNA loci	[77]
*Passiflora alata*, *P. watsoniana*	BAC-FISH with *Passiflora edulis* clones; Ty1-Copia and Ty3-Gypsy elements from *P. edulis*; FISH with 5S and 35S rDNA-targeting probes	Interspecific analyses of karyotype organisation; despite karyotype variability, no synteny breaks were observed in the chromosomal distribution of BACs and rDNA sites, except for an additional 35S rDNA locus on chromosome 3 of *P. watsoniana*; LTRs were uniformly dispersed, with occasional slight accumulation in proximal chromosome regions	[78]
*Phaseolus vulgaris*, *Vigna angularis*, *V. unguiculata*	BAC-FISH with *V. unguiculata* clones; oligo-FISH with CCP probes designed from the *P. vulgaris* genome sequence; FISH with a probe targeting 35S rDNA	Intergeneric analyses of karyotype organisation; first oligo-FISH CP in legumes; combination of BAC- and oligo-FISH resources, establishing a cytogenetic map of *V. angularis*; detection of CRs (translocations, inversions); chromosomes 2 and 3 identified as hotspots for CRs and de novo centromere formation	[79]
*Phaseolus vulgaris*, *Vigna unguiculata*	Oligo-FISH with barcode probes designed from the *Vigna unguiculata* genome sequence	Intergeneric analyses of karyotype organisation; in silico integration of previously established BAC-based chromosome-specific and rDNA markers with a newly developed oligo-FISH-based chromosome identification system; alignment of cytogenetic data with genome sequence data for representatives of two distinct genera; confirmation of known and detection of novel CRs (translocations, peri- and paracentric inversions)	[104]
Phaseoleae (Fabaceae): *Phaseolus vulgaris*, *Vigna unguiculata*, *Lablab purpureus*, *Macroptilium atropurpureum*	Oligo-FISH with painting probes specific to *Pv*2 and *Pv*3 chromosomes of *P. vulgaris*	Intergeneric analyses of karyotype organisation; inference of basic chromosome number and number of genomic blocks in the APK; the translocation between APK2 and APK3 is exclusive to *Phaseolus*, as chromosomes 2 and 3 of *L. purpureus* and *M. atropurpureum* resemble the orthologous chromosomes of *V. unguiculata* and are more closely related to the APK	[103]
*Phaseolus acutifolius*, *P. coccineus*, *P. dumosus*, *P. filiformis*, *P. leptostachyus*, *P. lunatus*, *P. macvaughii*, *P. microcarpus*, *P. vulgaris*, *P. vulgaris*	CCP with oligo probes designed from the *Vigna unguiculata* genome sequence and oligo probes specific for chromosomes 2 and 3 of *P. vulgaris*; FISH with 5S and 35S rDNA-targeting probes and the CentPv1 repeat probe	Karyotype evolution in the genus *Phaseolus*; detection of chromosomal rearrangements (translocations, inversions, duplications, deletions), primarily in species of the Leptostachyus group; *P. leptostachyus* experienced rapid genome reshuffling without whole-genome duplication, resulting in a reduction of its chromosome number from 11 to 10 pairs	[101]
*Vigna* subgenera: *Vigna*; *Plectrotropis*, *Haydonia*, *Lasiospron*, *Ceratotropis*	CCP with oligo probes specific for chromosomes *Pv*2 and *Pv*3 of *Phaseolus vulgaris*; barcode oligo probes designed from the *Vigna unguiculata* genome sequence; BAC clones derived from *P. vulgaris* and *V. unguiculata*; FISH with 5S and 35S rDNA-targeting probes	Karyotype evolution in the genus *Vigna*; chromosome identification across eight taxa; macrosynteny observed for chromosomes 2, 3, 4, 6, 7, 8, 9 and 10 in all taxa except *V. vexillata*, which possesses the most divergent karyotype; only minor differences in painting patterns observed among the subgenera	[81]
*Vigna lasicarpa*, *V. unguiculata*	CCP with oligo probes specific for chromosomes *Pv*1, *Pv*2, *Pv*3, and *Pv*5 of *Phaseolus vulgaris*; barcode oligo probes designed from the *Vigna unguiculata* reference genome; BAC clones derived from *V. unguiculata* and *P. vulgaris*; FISH with 5S and 35S rDNA-targeting probes and the T_3_AG_3_ telomeric repeat	Karyotype evolution in the genus *Vigna*; demonstration of conserved oligo-FISH patterns on chromosomes 2, 6, 8, 10 and 11 between *V. unguiculata* and *V. lasicarpa*; paracentric inversions in *Vla3* and *Vla9*; descending dysploidy in *V. lasicarpa* driven by end-to-end fusion of homoeologous chromosomes 5 and 7	[82]
*Lupinus angustifolius*, *L. cryptanthus*, *L. micranthus*, *L. cosentinii*, *L. pilosus*	BAC-FISH with *L. angustifolius* clones; oligo-FISH with probes specific for chromosome Lang06 of *L. angustifolius*	Karyotype evolution among five wild *Lupinus* species; demonstration of putative CRs within the Lang06 region, altering synteny and associated with speciation	[80]
*Cicer arietinum*	Oligo-FISH with chromosome-specific probes designed from the *C. arietinum* kabuli morphotype genome sequence; BAC-FISH with single-copy clones from the desi morphotype of *C. arietinum*; FISH with 5S and 35S rDNA-targeting probes and the T_3_AG_3_ telomeric repeat	Comparative analysis of closely related chickpea genotypes; individual chromosome identification and karyotype development; identification of CRs contributing to genome diversification among chickpea cultivars	[58]
*Tripidium arundinaceum*	Oligo-FISH with maize-derived painting probes; FISH with 5S and 35S rDNA-targeting probes	Chromosome identification and karyotyping in *T. arundinaceum* (sugarcane relative), effective despite 18 MY divergence from maize; conserved synteny with sorghum over 9 MYs	[100]
*Saccharum* complex *(Erianthus fulvus*, *E. rockii*, *Miscanthus sinensis*, *Narenga porphyrocoma*, *Saccharum officinarum*, *S. spontaneum*, *S. robustum*)	Oligo-FISH with painting probes designed from the *S. officinarum* genome sequence; FISH with 5S and 35S rDNA-targeting probes	Development of a set of 10 chromosome-specific landmarks effective for comparative karyotype analysis within the *Saccharum* complex; detection of CRs and novel cytotypes; chromosome fusions are common in various polyploids of the complex and alter the basic chromosome numbers	[90]
*Aegilops markgrafii*, *Ae. tauschii*, *Ae. umbellulata*, *Ae. uniaristata*, *Ae. speltoides*, *Triticum aestivum*	Oligo-FISH with D genome chromosome-specific painting probes designed from the *Triticum aestivum* genome sequence	Identification of the D genome in diploid (*Aegilops*) and polyploid (*T. aestivum*) species; detection of translocations involving D chromosomes, including two novel translocations (3D–7D and 4D–5D–7D) in three *Ae. tauschii* accessions; determination of the precise positions of chromosomal breakpoints in *Ae. tauschii* accessions; painting probes produce signals in four different genomes (U, C, M, N); CRs were identified in *Ae. umbellulata*, *Ae. markgrafii*, and *Ae. uniaristata*	[88]
Triticeae (Poaceae) genera: *Aegilops*, *Campeiostachys*, *Dasypyrum*, *Elymus*, *Hordeum*, *Roegneria*, *Thinopyrum*	Oligo-FISH with painting probes specific for chromosomes 1St to 7St, based on the *Pseudoroegneria libanotica* and *Triticum* *aestivum* reference genomes	Analysis of Triticeae karyotype organisation; identification of individual chromosomes of the St genome; conservation of St chromosomes in St-containing Triticeae representatives; weak St hybridisation signals observed in Y-genome chromosomes suggest an origin from the St genome	[84]
*Thinopyrum elongatum*, *Th. bessarabicum* wheat–tetraploid *Th. elongatum* substitution lines, *Triticum durum–Th. elongatum* amphidiploid	Oligo-FISH with painting probes designed from the *Th. elongatum* and *Triticum aestivum* genome sequences; tandem-repeat oligo probes pSc119.2 and pTa535	Development of a complete set of the E-genome painting probes to facilitate the detection of alien material in wheat breeding; chromosome identification; *Th. bessarabicum* (2x) shows a close genetic relationship with diploid *Th. elongatum*; five of the seven E-genome chromosomes exhibit complete synteny in both diploids, except for a reciprocal translocation between 4E and 5E^b^; a reciprocal translocation between 5E and 7E is present in one of the diploid *Th. elongatum* accessions	[83]
*Avena eriantha*, *A. fatua*, *A. nuda*, *A. sativa*, *A. ventricosa*, *A. wiestii*	Oligo-FISH with painting probes based on syntenic regions between wheat and barley; tandem-repeat oligo probes	Comparative karyotyping of eleven hexaploid and diploid *Avena* accessions; a high-resolution standard karyotype of *A. sativa* was established based on distinct FISH signals from multiple oligo probes	[86]
*Elymus dahuricus*, *Hordeum vulgare*	Oligo-FISH with painting probes based on syntenic regions between wheat and barley; oligo-FISH with repeat-based probes: pSc119.2, pTa535, Po5, 7E-716, 7E-599, 5S rDNA, 18S rDNA, 3A1, 13-J1011, 7E-744, d01-135, Ae334, and (GAA)_7_; GISH using *Pseudoroegneria spicata* gDNA; CENH3 immunolocalisation	Establishment of a universal karyotyping nomenclature system for *E. dahuricus*; precise determination of the linkage groups and sub-genomes of individual chromosomes; detection of a novel intergenomic rearrangement between the 2H and 5Y chromosomes in this allopolyploid	[85]
*Rhynchospora* (Cyperaceae): representative species from sections: *Albae*, *Dichromena*, *Cephalotae*, *Pauciflorae*, *Polycephalae*, *Pseudocapitatae*, *Tenues*	Oligo-FISH with two barcode probes (Rbv-I and Rbv-II) designed from the *R. breviuscula* genome sequence	Investigation of karyotype evolution and chromosomal variations in highly dynamic holocentric karyotypes; identification of all chromosomes in *R. breviuscula*; probes mapped in 13 other *Rhynchospora* representatives reveal CRs, including fusions, fissions, inversions, and translocations, as well as whole-genome duplication in *R. pubera*	[87]
*Musa acuminata*, *M. balbisiana*: wild subspecies, cultivars, and hybrids	Oligo-FISH with chromosome-specific probes designed using the *M. acuminata* genome sequence	Comparative karyotype analysis; detection of numerous accession-specific chromosome translocations correlated with banana speciation; demonstration of the complexity of banana genome evolution; identification of putative progenitors of banana cultivars	[60,89]
*Elaeis guineensis*, *E. oleifera*, *Cocos nucifera*, *Phoenix dactylifera*	Oligo-FISH with chromosome-specific probes designed using the *E. guineensis* genome sequence; FISH with a 5S rDNA-targeting probe	Establishment of a reference karyotype for *E. guineensis* and *E. oleifera*, identification of homoeologous regions in related species	[91]
*Citrus maxima*, *C. medica*, *C. mangshanensis*, *C. reticulata*, *Microcitrus australasica*, *Poncirus trifoliata*	Oligo-FISH with painting probes designed using the *Citrus maxima* genome; FISH with the 180 bp satellite repeat and with probes targeting 5S and 35S rDNA	Identification of all chromosomes in the studied species; complete chromosomal synteny was observed among six *Citrus* species over approximately 9 MYs of divergence, with no interchromosomal rearrangements identified in any species	[92]
*Fragaria*	Oligo-FISH with painting probes designed using the *Fragaria vesca* genome; FISH with 5S and 45S rDNA-targeting probes	Identification of individual chromosomes in 11 *Fragaria* representatives with different ploidy levels; comparative karyotyping; *Fragaria* species exhibit conserved karyotypes, with no interchromosomal rearrangements observed; differences in rDNA loci organisation patterns were found among polyploids; variations in signal intensity of oligo probes among homologous chromosomes in *Fragaria* polyploids provides new insights into their origins	[93]
*Ipomoea*	Oligo-FISH with barcode probes designed using *I. nil*; FISH with 5S and 35S rDNA-targeting probes	Comparative chromosome analysis in *I. batatas* and its wild relatives with different ploidy levels; *I. trifida* is the most closely related diploid to *I. batatas*; providing cytogenetic evidence for the segmental allopolyploid hypothesis of sweet potato origin	[94]
Sixteen diploid *Ipomoea* species representing all seven minor clades	Oligo-FISH with painting probes designed using *I. nil* chromosomes 7 and 15; FISH with 5S and 35S rDNA-targeting probes	Comparative chromosome analysis across the genus; significant cytogenetic divergence between 2n = 28 and 2n = 30 species, questioning molecular phylogeny-based classifications that group them into the same clade; significant interspecific variation in rDNA loci distribution complements CCP-based analyses	[99]
*Cucumis sativus* var. *sativus*, *C. sativus* var. *hardwickii*, *C. hystrix*, *C. melo*, *C. metuliferus*, *C. subsericeus*, *C. dipsaceus*, *C. zeyheri*, *C. anguria*	Oligo-FISH with painting probes designed using the *C. sativus* genome sequence	Designing chromosome painting oligo probe libraries; reconstruction of the ancestral karyotype for the genus; comparative analysis reveals the genome structure of all studied species and complex CRs that occurred during *Cucumis* karyotype evolution; compared to African species, Asian-origin species possess genomes that are highly reshuffled due to large-scale inversions, centromere repositioning, and chromothripsis-like events	[97,98]
*Glycyrrhiza eglandulosa*, *G. glanndulosa*, *G. eurycarpa*, *G. glabra*, *G. inflata*, *G. prostrata*, *G. uralensis*	Oligo-FISH with painting probes designed using the *Glycyrrhiza uralensis* genome sequence; FISH with 5S and 45S rDNA-targeting probes	Chromosome identification in *G. uralensis* and its relatives; exceptionally conserved chromosomal synteny was observed after 3–12 MYs of divergence, with no cytologically visible interchromosomal rearrangements detected by CP	[95]
*Gossypium hirsutum*	Oligo-FISH with probes designed using the *Gossypium hirsutum* genome sequence and targeting single and multiple chromosomes; FISH with oligo probes targeting telomeric sites and 5S and 45S rDNA loci	Developing robust markers for chromosome identification in previously intractable species	[96]
*Pulmonaria officinalis* group (*P. obscura*, *P. officinalis* s. str.)	Multicolour FISH with tandem-repeat probes (five newly identified satellite DNAs, 5S and 45S rDNA)	Designing a new set of chromosome-specific landmarks; comparative karyotyping; chromosome structure in *P. officinalis* s. str. is more variable than in *P. obscura*; confirmation of the hybrid status of 2n = 15 putative hybrids collected from mixed populations of *P. obscura* and *P. officinalis* s. str.	[105]
*Silene latifolia*, *S. dioica*, *S. vulgaris*, *S. maritima*	Comparative oligo-FISH with an X-chromosome-scaffold-originated probe designed from the *S. latifolia* genome sequence; *Silene* STAR-C centromeric and X43.1 repeat DNA probes	Development of a more robust probe for visualising *Silene* sex chromosomes than any previous markers, and investigation of their evolution; the hybridisation of this probe to the short arms of several autosomes in *S. vulgaris* and *S. maritima* suggests that extensive CRs played a role in the evolution of *Silene* sex chromosomes	[102]
Twelve wild representatives of the *Hordeum* genus	Comparative BAC-FISH with the Hbog_46L9 clone from *H. bogdanii*, which contains a *Panicum*-derived chromosomal segment; FISH with a 45S rDNA-targeting probe	Demonstration of horizontal gene transfer by identifying and characterising a foreign chromosomal segment from a *Panicum*-like donor in wild barley species, highlighting its evolution and dynamics within host genomes	[106]
Nearly 30 species of the *Onobrychis* genus	FISH with 5S and 35S rDNA-targeting probes	Determination of the number and chromosomal distribution of rDNA loci, along with the identification of selected chromosomes; demonstration of polymorphism in rDNA chromosomal patterns in diploids, contrasted with its absence in polyploids; inference of the ancestral basic chromosome number, rDNA loci counts, and mechanisms such as polyploidisation and descending dysploidy that have shaped chromosome number evolution in the genus	[107]

## 3. Chromatin and Interphase Nuclear Organisation

Plant nuclear DNA is arranged into chromatin, which not only carries genetic information but also plays crucial roles in processes such as DNA replication, repair, and transcriptional regulation. The three-dimensional (3D) organisation, epigenetic status, and cis- and trans-interactions of chromatin within the nucleus are essential for its function as well as dynamic responses to developmental signals and environmental cues (4D genomics) [108]. Significant progress has been made in deciphering spatial nuclear architecture in plants, largely due to the development of chromosome conformation capture (3C) techniques, such as Hi-C (high-throughput chromatin conformation capture), ChIA-PET (chromatin interaction analysis by paired-end tag sequencing), and HiChIP (in situ Hi-C followed by chromatin immunoprecipitation) (for recent reviews, see [109,110,111]). Furthermore, a broad array of FISH-based approaches, including 3D-FISH and oligo-FISH with painting probes, combined with super-resolution microscopy, have provided valuable insights into how the spatial arrangement of chromosome territories (CTs) influences gene expression [66,112] (section II of Figure 2). Although the existence of CTs in plants was demonstrated some time ago, first in arabidopsis [19] and subsequently in other species (e.g., [24,113]), the mechanisms underlying their establishment and maintenance remain poorly characterised. Recent discoveries related to these processes are summarised in Table 2 and outlined below. Attachment of chromatin to the nuclear envelope, mediated by lamin-like nuclear matrix constituent proteins such as CRWN1 and CRWN4, appears to be a key factor [63,114] (Figure 2G). In addition to CRWN proteins, condensin complexes also contribute to interphase chromatin architecture. Specifically, the role of arabidopsis CAP-D3 in centromere and telomere positioning has been reported [115]. Studies using *cap-d3* mutants demonstrate that arabidopsis forms distinct condensin I and II complexes, and that CAP-D3 facilitates the spatial separation of chromocenters without affecting global DNA or histone methylation patterns [62] (Figure 2F).

The interphase nuclei of arabidopsis show a rosette-like CC-loop organisation with telomeres clustering near the nucleolus, whereas related crucifer species with larger genomes display Rabl-like or dispersed configurations. This suggests that genomic properties, such as genome size and degree of longitudinal chromosome compartmentalisation, rather than phylogenetic position, determine interphase nuclear organisation in crucifer genomes [116]. Similarly, stable chromosome positioning independent of genome size, as well as conserved DNA replication dynamics, have been observed across seven Poaceae species [117]. A Rabl-like chromosome configuration in interphase nuclei, revealed through 3D-FISH, was also detected in Limnanthaceae (Brassicales), a family closely related to arabidopsis [118]. In the monocot *Oryza sativa* (rice) most somatic cell nuclei lack Rabl configuration [119], contrasting with another monocot model plant, *Brachypodium*, which exhibits Rabl organisation in certain cell types [120]. A recent study on *O. sativa* interphase nuclei revealed tissue-specific chromatin architecture, with differences in condensation levels and CT arrangements between leaf and root cells. Chromosome positioning, CT volumes, and spatial associations were shown to depend on nucleolus size and the activity of the 45S rDNA locus, underscoring the connection with nucleolar architecture [61] (Figure 2D,E). The dynamic organisation of chromatin in relation to the cell cycle and developmental cues has also been investigated in *Hordeum vulgare* (barley). Distinct centromere and telomere arrangements observed between cycling and endoreduplicated nuclei in embryo and endosperm tissues indicate that the Rabl configuration is established and maintained through mitotic divisions, and that it is further influenced by tissue identity and seed developmental stage [121].

**Table 2:** A selection of recent research articles on molecular cytogenetic analyses of chromatin and interphase nuclear organisation.

Research Object	Research Approach	Aims and Main Findings	References
*Arabidopsis thaliana*, *Hordeum vulgare*	3D FISH using centromeric, 35S rDNA-targeting, telomeric, subtelomeric, and H5L-specific painting oligo probes; immunodetection of RNA polymerase II, ASY1, ZYP1, DMC1, HEI10, SSSU, and H3K27me3; visualisation of a GFP-tagged protein associated with the nuclear envelope; imaging performed using diffraction-limited confocal microscopy and super-resolution microscopy	A compendium of strategies to analyse the spatial distribution of nuclear and chromosomal signals from 3D image stacks	[66]
*Arabidopsis thaliana* (Col-0 and *ddm1-2*)	Image analysis using the semi-automatic ImageJ plug-in iCRAQ (https://github.com/gschivre/iCRAQ, accessed on 15 July 2025) and the DL-based tool Nucl.Eye.D (https://zenodo.org/records/7075507, accessed on 15 July 2025)	Detection and quantification of *A. thaliana* nuclear features using two segmentation methods: iCRAQ (semi-automated) and Nucl.Eye.D (deep learning), enabling precise analysis	[112]
*Populus trichocarpa*	3D-FISH on frozen root tip sections; FISH with a 45S rDNA-targeting probe and oligopainting probes for chromosomes 17 and 19	Improved signal quality compared to paraffin sections; chromosome-specific oligo probes enabled 3D analysis of chromosome territories; autosome pair 17 associated more frequently than sex chromosome 19	[113]
*Arabidopsis thaliana* WT and *crwn1-1*, *crwn4-1*, and *kaku4-2* mutants	BAC-FISH using clones specific to *A. thaliana* chromosomes 1 and 3	Plant chromatin organisation is flexible, adapting to developmental and environmental cues; under heat stress, the nuclear lamina disassembles, and chromatin domains relocate from the nuclear envelope to the inner nucleus while remaining associated with CRWN1; CRWN1 plays a key role in genome folding dynamics during stress	[63]
*Arabidopsis thaliana* Columbia-0 and *cap-d3* T-DNA insertion mutants	FISH using 180 bp centromeric repeat, 5S and 45S rDNA-targeting probes; immunostaining with antibodies against histone modifications H3K27me3, H3K9me1, H3K9me2, H3K4me3, H3K9ac, H3K14ac, H3K18ac or H3K9 + 14 + 18 + 23 + 27ac, and 5-methylcytosine	Evaluation of the role of CAP-D3 in interphase chromatin organisation and function; in *cap-d3* mutants, heterochromatic sequences show increased association, while nuclear size and the general histone and DNA methylation patterns remain unchanged	[62]
*Arabidopsis thaliana*, *Arabis cypria*, *Bunias orientalis*, *Cardamine amara*, *Descurainia preauxiana*, *Euclidium syriacum*, *Hesperis sylvestris*	2D and 3D FISH using centromere-specific oligo probes: pAL, ArCy1, CARCEN, HeSy1, and de novo identified 156-bp repeat of *D. preauxiana*; telomeric repeat and *A. thaliana* BAC clone T15P10 (AF167571) containing 35S rRNA genes	The CC-loop model in *Arabidopsis thaliana* links telomeres to the nucleolus; in crucifers, small genomes exhibit nucleolus-associated telomere clustering, whereas large genomes display a Rabl-like configuration or a dispersed chromosomal distribution	[116]
*Avena sativa*, *Brachypodium distachyon*, *Hordeum vulgare*, *Oryza sativa*, *Secale cereale*, *Triticum aestivum*, *Zea mays*	Nuclei sorting by flow cytometry; 5-ethynyl-2′-deoxyuridine labelling; FISH using a telomere oligo probe; centromere immunovisualisation with an antibody against OsCenH3 (rice centromeric histone H3 variant)	Conserved DNA replication dynamics and chromosome positioning across seven Poaceae species with varying genome sizes	[117]
Limnanthaceae, Brassicales	2D and 3D FISH using centromere-specific oligo probes, telomeric repeat, *Arabidopsis thaliana* BAC clone T15P10 (AF167571) containing 35S rRNA genes, and clone pCT4.2 (M65137) corresponding to the 5S rDNA repeat	Five chromosome pairs in the interphase nuclei of *Limnanthes* species adopt a Rabl-like configuration	[118]
*Oryza sativa*	Oligo-FISH using painting probes specific for chromosome 9 and the S and L arms of chromosome 2; FISH with probes targeting centromeric, telomeric, and 45S rDNA sites	Six chromosome territory (CT) configurations were identified in *O. sativa* root meristematic nuclei and four in leaf nuclei, showing variations in CT volume and association frequency; the association of chromosome 9 CTs was influenced by 45S rDNA activity, linking nuclear organisation to the position and size of the nucleolus	[61]
*Hordeum vulgare*	Nuclei sorting by flow cytometry; oligo-FISH using a barley centromere-specific probe; FISH with telomeric repeats, 5S and 45S rDNA-targeting probes; immunostaining with antibodies against *H. vulgare* CENH3	Analysis of nuclear morphology and chromosome organisation in cycling and endoreduplicated nuclei isolated from embryo and endosperm tissues of developing barley seeds; endoreduplicated nuclei exhibit irregular shapes, show reduced sister chromatid cohesion at 5S rDNA loci, and decreased CENH3 levels; progressive endoreduplication leads to intermingling centromeres and telomeres	[121]

## 4. Chromosome Structure

The metaphase chromosome represents the structural state in which chromatin reaches its highest level of compaction. Recent studies examining plant chromatin organisation throughout the cell cycle, as well as the architecture of metaphase chromosomes, are presented in Table 3, section III of Figure 2, and briefly outlined below. Two models have been proposed to describe the higher-order organisation of metaphase chromosomes: helical and non-helical. Recent studies combining Hi-C analysis, biopolymer modelling, and structured illumination microscopy have confirmed a helical organisation of barley metaphase chromosomes, consistent with observations in chicken and HeLa cells [122]. Imaging techniques such as transmission electron microscopy [123], scanning electron microscopy [124], and super-resolution microscopy [125] (Figure 2J) have been employed to validate the presence of this helical chromatin structure. Among these techniques, super-resolution microscopy enables the localisation of topoisomerase (Topo) proteins along chromosomes [126]. The localisation and distribution of Topo II have also been investigated using high-voltage transmission electron microscopy and ultra-high-voltage transmission electron microscopy combined with immunogold labelling [127]. Advances in the analysis of plant chromosome structure using various electron microscopy techniques have recently been reviewed by Ohmido et al. [128].

Our understanding of mitotic dynamics has traditionally relied on fixed-sample techniques, which limit insights into the kinetics of chromatin, nucleoli, microtubules, and the duration of individual mitotic stages. However, studies employing fluorescent protein translational fusion lines in barley, combined with confocal microscopy, have yielded valuable insights into nuclear organisation and mitotic dynamics in living root meristematic cells [129]. During eukaryotic cell division, each daughter cell must inherit a balanced chromosome complement, a process critically dependent on properly functioning centromeres. Centromeres assemble kinetochore complexes essential for spindle microtubule attachment. Comprehensive reviews on the structure, function, and evolution of plant centromeres have recently been published [130,131,132]. In plants, kinetochore assembly sites are marked by the presence of the centromeric histone H3 variant (CENH3), which can be localised using specific antibodies. Recent studies have successfully identified and localised this protein in many species, including *Agave tequilana* relatives [133], *Prionium serratum* [65] (Figure 2I), *Chionographis japonica* [134], *Cuscuta* spp. [135], and *Gossypium* species [136]. Unlike canonical histones, CENH3 evolves rapidly, and its N-terminal tail exhibits high variability even among closely related species. Due to this variability, antibodies targeting conserved domains of outer kinetochore proteins, such as KNL1 and NDC80, offer greater versatility for centromere immunolabeling across diverse plant taxa [137].

Most plants possess monocentric chromosomes, characterised by centromeres localised to a single chromosomal region. However, numerous species have independently evolved holocentric chromosomes, where spindle microtubules attach along the entire chromosome length [138]. A comprehensive analysis of holocentric chromosome architecture, incorporating oligo-FISH with satellite repeats, immunostaining of CENH3 and kinetochore proteins, and histone modification profiling, has recently been conducted in *Chionographis japonica* [134] and *Luzula sylvatica* [139]. Another form of centromere organisation, known as metapolycentricity, has been observed in *Pisum* [64] (Figure 2H). These chromosomes are cytologically characterised by extended primary constrictions and multiple discrete domains of CENH3 chromatin. A transition from monocentricity to holocentricity has been reported within the genus *Cuscuta*, where the kinetochore protein gene KNL2 is absent, CENH3 is enriched in heterochromatic regions, and microtubule attachment occurs along the full chromosomal length [135].

**Table 3:** A selection of recent research articles addressing various aspects of chromosome structure from a cytomolecular perspective.

Research Object	Research Approach	Aims and Main Findings	References
*Hordeum vulgare*	Immunostaining with antibodies against Topo IIα and grass CENH3 (centromeric histone H3 variant); structured illumination microscopy (SIM) and photoactivated localisation microscopy	Topo IIα is dispersed along chromosome arms but accumulates at centromeres, telomeres, and NORs; at centromeres, Topo IIα intermingles with CENH3-containing chromatin	[126]
*Hordeum vulgare*	Oligo-FISH with probes specific to the 5HL chromosome; 5-ethynyl-2′-deoxyuridine (EdU) labelling; analysis of purified metaphase chromosomes; biopolymer modelling; spatial SIM of large fluorescently labelled chromosome segments	Direct differential visualisation of a condensed chromatin fibre confirms the helical model; revealing chromonemas—helically wound, 400-nm-thick chromatin threads that form the chromatids of mitotic chromosomes	[122]
*Agave tequilana*, *Hesperaloe funifera*, *H. parviflora*, *Hesperoyucca whipplei*, *Yucca carnerosana*, *Y. constricta*, *Y. elata*	Immunostaining with antibodies against agavoid CENH3; 3D super-resolution microscopy; scaling relationship of kinetochore size to chromosome size in the karyotype	A positive intra-karyotype relationship between kinetochore and chromosome size, similar to that observed in other eucaryotes; the scaling of total kinetochore size to genome size may originate from the mechanics of cell division	[133]
*Prionium serratum*	Immunostaining with antibodies against *P. serratum* CENH3, α-tubulin, histone H3S28ph, and histone H2A120ph	*P. serratum* exhibits a monocentric chromosome organisation, in contrast to the holocentricity observed in other species of the Cyperid clade (Thurniceae-Juncaceae-Cyperaceae)	[65]
*Chionographis japonica*	Oligo-FISH with probes for *C. japonica* satellite repeats (centromeric Chio1 and Chio2), LTR transposable elements, telomeric repeat, and 45S rDNA FISH; immunostaining with antibodies against *C. japonica* CENH3, MIS12, NDC80, and α-tubulin, as well as histone modifications including H3K4me2, H3K9me2, H3S10ph, H3S28ph, H3T3ph, and H2AT120ph; EdU labelling	Holocentric chromatids of *C. japonica* consist of 7–11 evenly spaced, megabase-sized centromere-specific histone H3-positive units, which contain satellite arrays of 23- and 28-bp-long monomers; the large-scale eu- and heterochromatin arrangement differs between *C. japonica* and other known holocentric species	[134]
*Luzula sylvatica*	Oligo-FISH with probes for satellite repeats *Lusy1* and *Lusy2*; immunostaining with antibodies against *L. elegans* CENH3, KNL1, NDC80, and α-tubulin	*L. sylvatica* holocentromeres are predominantly associated with two satellite DNA repeats, *Lusy1* and *Lusy2*, while CENH3 also binds to satellite-free gene-poor regions; *Lusy1* plays a crucial role in centromere function across most *Luzula* species; holocentric chromosomes in *Luzula* may have originated from chromosome fusions of ancestral monocentric chromosomes and the expansion of CENH3-associated satDNA	[139]
*Pisum sativum* and related representatives of the Fabeae tribe (*Pisum*, *Lathyrus*, *Vicia*)	Oligo-FISH with painting probes PS6, for *P. sativium* chromosome 6, satDNA-based FabTR probes; immunostaining with anti-CENH3 antibodies	Assembly and analysis of a 177.6 Mb region of *P. sativum* chromosome 6 which includes 81.6 Mb centromere region (CEN6) and adjacent segments of both chromosome arms; three satellite repeats were associated with CENH3-enriched chromatin, while five others were not; comparative analysis revealed that the evolution of metapolycentromeres is driven by the expansion of centromeric chromatin into neighbouring chromosomal regions, accompanied by the accumulation of novel satellite repeats, which are complemented by CRs in some species	[64]
*Cuscuta europaea*, *C. epithymum*, *C. australis*, *C. campestris*, *C. reflexa*	Immunostaining with antibodies against *Cuscuta* CENH3, KNL1 and KLN2, CENP-C, MIS12, NDC80, BUB3;1/2, borealin, and α-tubulin	The transition from monocentricity to holocentricity in the genus *Cuscuta* was accompanied by dramatic changes in the kinetochore, including the loss of centromeric localisation of CENH3, CENP-C, KNL1, MIS12, and NDC80 proteins, as well as and the degeneration of the spindle assembly checkpoint (SAC); these changes indicate that holocentric *Cuscuta* species have lost the ability to form a standard kinetochore and no longer utilise the SAC to regulate microtubule attachment to chromosomes	[135]
*Arabidopsis thaliana*, *Chionographis japonica*, *Cuscuta reflexa*, *Dionaea muscipula*, *Drosera capensis*, *Juncus effusus*, *Luzula nivea*, *Nelumbo nucifera*, *Nymphaea alba*, *Ocimum basilicum*, *Picea abies*, *Pisum sativum*, *Raphanus sativus*, *Rhynchospora pubera*, *Triticum aestivum*	Immunostaining with antibodies against KNL1, NDC80, and α-tubulin	The KNL1 and NDC80 antibodies effectively labelled centromeres in condensed chromosomes during cell division, as well as the interphase nuclei of most species tested; KNL1 and NDC80 antibodies are better suited for immunolabeling centromeres than CENH3 antibodies, providing greater versatility across different plant species and enabling the study of centromere organisation in non-model species	[137]
*Gossypium anomalum*, *G. arboreum*, *G. hirsutum*, *G. raimondii*	Immunostaining with antibodies against cotton CENH3; FISH with probes for centromeric repeats of *G. anomalum*	Characterisation of *G. anomalum* centromeric sequences using chromatin immunoprecipitation against CENH3 antibodies; *G. anomalum* centromeres contained only retrotransposon-like repeats and lacked long arrays of satellite DNA	[136]
*Petunia axillaris* subsp. *axillaris*, *P. axillaris* subsp. *parodi*, *P. integrifolia* subsp. *inflata*, *P. × hybrida*	FISH with PSAT1, PSAT3, PSAT4, PSAT5, PSAT6, and PSAT7 satellite repeat probes	Seven repeat families (PSAT1, PSAT3, PSAT4, PSAT5, PSAT6, PSAT7, PSAT8) exhibited high sequence similarity and organisation across the four *Petunia* genomes; these repeat families occupy distinct chromosomal niches, differing in copy number and organisation	[140]
*Rosa arvensis*, *R. multiflora*, *R. rugosa*, *R. majalis*, *R. nitida*, *R. persica*	FISH with 18S rDNA and probes derived from the 5S rDNA genic region, as well as 5S_B and 5S_A IGS subregions	Locus-specific probes determined the number and chromosomal position of 5S rDNA families; two major 5S rDNA families (5S_A and 5S_B) were identified in *Rosa* diploids and polyploids; the 5S_B family often co-localised with 35S rDNA at NORs, while such co-localisation of the 5S_A family was rare	[141]
41 woody plants representing 37 species and 27 genera, and 18 families, *Zea mays*	Oligo-FISH with the (AG_3_T_3_)_3_ probe	The AG_3_T_3_ sequence was observed at chromosome termini in 38 plants; its non-telomeric signals were detected in 23 plants, being particularly abundant in *Chimonanthus campanulatus*	[142]
*Hordeum vulgare* fluorescent marker lines	Time-lapse confocal microscopy imaging	Development of unique materials enabling detailed live-cell imaging of mitosis and cytokinesis; determination of the duration of mitosis and its stages in barley; demonstration that chromosome condensation in barley often precedes the mitotic preprophase	[129]
*Hordeum vulgare*	Electron tomography	Dissecting the 3D higher-order structure of metaphase chromosomes using a thin carbon film in electron tomography revealed periodic structures with a 300–400 nm pitch along the barley chromosome axis; their periodicity was twice that of the corresponding structures found in human chromosomes	[123]
*Hordeum vulgare*	Immunogold labelling; immunostaining with barley-specific antibody against Topo II; high-voltage transmission electron microscopy (HVTEM) and ultra-high-voltage transmission electron microscopy (UHVTEM)	HVTEM and UHVTEM combined with immunogold labelling are effective for detecting structural proteins such as Topo II; Topo II molecules are distributed along barley chromosomes in a non-specific pattern, with distinct accumulation at the chromosome termini, nucleolus organiser, and centromeric regions	[127]
*Hordeum vulgare*	Immunostaining with barley-specific antibodies against Topo IIα applied to flow-sorted chromosomes; sub-diffraction variants of fluorescence super-resolution microscopy, such as structured illumination, stimulated emission depletion, and single-molecule localisation microscopy	Protein imaging in barley metaphase chromosomes: comparing selected super-resolution approaches with conventional wide-field and confocal microscopy in terms of mapping resolution and accuracy	[125]
*Hordeum vulgare*	Scanning electron microscopy (SEM)	Investigating the role of calcium ions (Ca^2+^) in the chromosome structure of barley; BAPTA treatment led to a less condensed, dispersed chromosome structure due to Ca^2+^ chelation; high-resolution SEM provided detailed visualisation of chromosome ultrastructure under different calcium ion conditions	[124]
A human–*Arabidopsis thaliana* hybrid cell line containing a neo-chromosome	FISH using fifteen probes targeting *A. thaliana* single-copy genome regions, *A. thaliana* centromere repeat (Atcen, 180 bp), and telomeric short repeats of human (T_2_AG_3_) and *A. thaliana* (T_3_AG_3_)	The structure and function of plant and animal chromosomes are largely conserved, enabling the creation of a human–*A. thaliana* hybrid cell line; a neo-chromosome was formed by inserting segments of *A. thaliana* chromosomes 2–5 into human chromosome 15; the neo-chromosome contained *A. thaliana* centromeric repeats and human telomeres; however the *A. thaliana* centromere was not functional; most *A. thaliana* DNA was eliminated during culture	[143]
*Secale cereale*, *Triticum aestivum*, *Aegilops speltoides*	FISH with 5S rDNA, *S. cereale* genome-specific repeat Revolver, B-chromosome-specific repeats (D1100, E3900, Sc9c130, Sc26c38), and the *DCR28* gene family	Based on a newly assembled ~430 Mb rye B chromosome pseudomolecule, five candidate genes were identified as trans-acting moderators influencing targeted B chromosome nondisjunction during the first pollen mitosis; among them is *DCR28*, a microtubule-associated gene; the *DCR28* gene family appears to be neo-functionalised and is uniquely highly expressed during the first pollen mitosis in rye	[144]
*Sorghum purpureosericeum*	FISH on embryo sections, pollen grains, and meiocytes using the B-specific repeat CL135 and the centromeric probe CL29	B chromosome occurrence is tissue- and organ-specific, primarily due to extensive elimination during embryo development, which continues throughout plant growth; accumulation of B chromosomes results either from nondisjunction during the first pollen mitosis or from additional nuclear divisions during pollen development	[145]
*Aegilops speltoides*	FISH with repetitive DNA probes Spelt1, Spelt52, pSc119.2, pTa71 (45S rDNA), As5SDNAE (5S rDNA), CCS1 (centromeric), and T_3_AG_3_ (telomeric)	Ectopic associations between B and A chromosomes were observed, along with cell-specific rearrangements of B chromosomes in both mitosis and microgametogenesis; the copy numbers of selected transposable elements and tandem repeats varied with genotype and tissue type, but were unaffected by the presence or absence of B chromosomes	[146]

Supernumerary B chromosomes are often distinguishable from the standard A chromosomes within the karyotype. Their enigmatic role, non-Mendelian behaviour during meiosis, and frequent irregular segregation in postmeiotic mitoses continue to make them compelling subjects of study (reviewed by [147]). For example, Chen et al. [144] employed a newly assembled *Secale cereale* (rye) B chromosome pseudomolecule to identify five candidate genes acting as trans-acting regulators of B chromosome drive in developing pollen. These included the *DCR28* gene family, which encodes a protein associated with cell division and was also identified on *Aegilops speltoides* B chromosomes. In contrast, studies in *Sorghum purpureosericeum* demonstrate B chromosome instability during plant ontogenesis, with their elimination occurring primarily during embryo development. This leads to distribution patterns restricted to specific tissues or organs, which are largely preserved post-embryonically [145]. Interestingly, in *Ae. speltoides*, the presence or absence of B chromosomes did not appear to influence the copy number dynamics of mobile elements and tandem repeats, despite observed ectopic associations between supernumerary and standard chromosomes. This complicates efforts to disentangle the specific roles of B chromosomes from other factors affecting nuclear genome integrity and dynamics [146]. However, recent findings in maize demonstrate that such distinctive roles do exist., For example, they influence the distribution and characteristics of R-loops on A chromosomes in a tissue-specific manner, thereby affecting various cellular processes, including gene expression [148].

## 5. Natural and Induced Hybridisation and Polyploidy

Polyploidisation, the multiplication of complete chromosome sets, is a widespread phenomenon and plays a crucial role in angiosperm speciation, adaptation and evolution [149,150,151]. Cytomolecular approaches using FISH with various sequences as probes, particularly gDNA, have long proved effective in studying plant polyploids by enabling the visualisation of chromosome sets contributed by their evolutionary parents (e.g., [152,153]). In some cases, they have provided the first, yet robust, indication of the polyploid nature of newly discovered species (e.g., [154]). Recent cytomolecular studies have further yielded valuable insights into taxonomic delineation and genome evolution across diverse taxa (see Table 4 and section IV of Figure 2). For example, they have clarified the complex origins of *Hieracium* allopolyploids [155], revealed interspecific relationships and non-homologous chromosomal rearrangements in Triticeae species [36], and contributed to tracing the evolution of the *Camelina* genus by identifying a *C. neglecta*-like genome (*C. intermedia*) as a potential ancestor of *C. sativa* [156]. Furthermore, GISH-based analyses have shed light on hybridisation events between the native *Opuntia rioplatensis* and the introduced North American species *O. ficus-indica*, which likely led to the formation of the taxon described as *O. cristalensis* [157]. The same approach was employed by Shimomai et al. [158] to demonstrate how polyploidisation in *Commelina* contributes to enhanced survival in urban environments.

In addition to analyses of naturally evolved polyploids, molecular cytogenetic methods have proven invaluable for studying synthetic auto- and allopolyploids. These, apart from their usefulness in breeding, serve as informative model systems for investigating early genome evolution following polyploidisation [159,160]. The formation of synthetic polyploids can trigger various genomic responses, including non-homologous chromosome pairing, translocations, deletions and other structural rearrangements [152,159]. Variation in chromosome number, accompanied by chromosome translocations, has been observed in synthetic *Triticum turgidum* × *Aegilops umbellulata* hybrids [161].

**Table 4:** A selection of recent research articles on cytomolecular analyses related to natural hybridisation and polyploidy.

Research Object	Research Approach	Aims and Main Findings	References
*Saccharum spontaneum*	Oligo-FISH with haplotypic probes of *S. spontaneum* specific to chromosomes 8A, 8B, 8C, and 8D	Whole genome duplications in autopolyploid sugarcane AP85–441; no chromosomal aberrations found between autotetraploid AP85–441 and its spontaneously doubled version indicate strict regulation of chromosome duplication	[162]
*Hieracium intybaceum*, *H pallidiflorum.* *H. picroides*, *H. prenanthoides*	GISH with gDNA of *H. intybaceum* and *H. prenanthoides*; FISH with 5S and 35S rDNA-targeting probes	One of the first multiapproach studies of apomictic *Hieracium* allopolyploids; multiple origins of hybridogenous *H. pallidiflorum* and *H. picroides* from the same diploid–polyploid parental species *H. intybaceum* and *H. prenanthoides*; new insight into the taxonomic delineation of the species	[155]
*Opuntia*	FISH with 5S and 35S rDNA-targeting probes	*O.* × *cristalensis* appears to be a hybrid between the native Argentine species *O. rioplatensis* and the North American introduced species *O. ficus-indica*; the number of 5S rDNA sites in *O.* × *cristalensis* reflects its ploidy level, whereas the number of 35S rDNA sites does not; probably the first documented case of hybridisation between North and South American *Opuntia* species	[157]
Triticeae	FISH with (1) single copy oligos associated with each of the A-, B-, and D-genome chromosomes of *Triticum aestivum*; (2) oligos from 1H to 7H chromosomes designed using *Hordeum vulgare* genome; (3) the conserved oligos based on a wheat reference genome; (4) Synt1 to Synt7 oligos from the syntenic region with > 96% homology in wheat-barley linkage groups; (5) Synt7SL barcoding oligos	Development of a chromosome-specific painting using oligo pools for large-genome Triticeae species; high-throughput karyotyping of Triticeae and some wheat-alien derivatives; tracking interspecific chromosome homologous relationships and non-homologous CRs	[36]
*Commelina benghalensis*, *C. communis* (Cc), *C. communis* f. *ciliata* (Ccfc)	GISH with gDNA of Ccfc	Investigation of the role of polyploidisation in the distribution and survival of Cc and its subspecies Ccfc across urban-rural gradients; urban areas were dominated by Cc, whereas both Cc and Ccfc coexisted in rural areas; polyploidy and an additional genome provide Cc with enhanced survival in urban environments	[158]
Synthetic *Triticum turgidum–Aegilops umbellulata* hybrids	GISH with gDNA of *Ae. umbellulata*; FISH with following probes: oligo-pTa-535 (pTa535), oligo-pSc119.2 (pSC119.2), oligo-pTa71 (pTa71—35S rDNA-targeting probe), and (AAC)_5_	Investigation of unreduced gamete formation mechanisms in *T. turgidum–Ae. umbellulata* triploid F1 hybrid crosses and the chromosome compositions in their F2 generations; chromosome numbers in F2 plants ranged from 35 to 43, with variations in chromosome loss/gain among genomes, chromosome loss was highest in the U genome; three types of chromosome translocations and polymorphic FISH karyotypes were identified	[161]
*Camelina intermedia*, *C. hispida*, *C. laxa*, *C. neglecta*, *C. sativa*	GISH with gDNA of *C. hispida*, *C. laxa*, *C. neglecta*, and *C. intermedia*; CCP with *Arabidopsis thaliana* BAC contigs as painting probes	The identification of the maternal genome of the allohexaploid *C. sativa*; a tetraploid *C. neglecta*-like genome (*C. intermedia*) is hypothesised to be the likely maternal ancestor of the *C. sativa* based on its high collinearity with two maternally inherited subgenomes; the study contributes to completing the image of the evolution of the *Camelina* genus	[156]
Synthetic and natural allotetraploid wheat hybrids	FISH with centromere-specific retrotransposon of wheat and the telomere repeat; multicolour GISH with gDNA of *Triticum urartu*, *Aegilops longissima* and *Ae. tauschii*	A series of nascent allotetraploid wheats from three diploid genomes (A, S*, and D) was synthesised; most progeny had consistent chromosome numbers, with each genome containing 14 chromosomes, suggesting stable chromosome number inheritance due to diploidisation; detected aneuploids have affected centromere pairing and clustering in early meiosis	[163]
Autopolyploid (4x, 8x, 10x) clones of *Saccharum* *spontaneum*	Oligo-FISH with painting probes specific for chromosomes 1 (Chr. 1), 7 (Chr. 7), and 8 (Chr. 8) of *S. spontaneum*	All clones showed stable, diploid-like chromosome behaviour during meiosis; in the 4x clone, two and two copies of Chr. 8 are of different size, and the pairing likely occurs between the homologs of similar size; considering high sequence similarity among Chr. 8 homologues, some unknown mechanisms are responsible for their peculiar pairing behaviour in the 4x clone	[67]
*Brachypodium hybridum*	FISH with 5S and 35S rDNA-targeting probes	Investigation of nucleolar dominance (ND) stability in *B. hybridum* genotype 3-7-2 compared to the reference genotype ABR113 revealed differences in tissue-specific expression; in ABR113, ND remained stable across all tissues, including primary and adventitious roots, leaves, and spikes; genotype 3-7-2 exhibited a strong upregulation of S-subgenome units in adventitious roots, but not in other tissues	[164]
*Brachypodium hybridum*, *B. distachyon*, *B. stacei*	FISH with 5S and 35S rDNA-targeting probes	Analysis of the structure, expression, and epigenetic landscape of 35S rDNA in allopolyploid *B. hybridum* and its diploid progenitors, *B. distachyon* and *B. stacei*; in *B. hybridum*, the copy number of *B. stacei* 35S rDNA homoeologues was reduced, accompanied by their transcriptional inactivation; DNA methylation played a role in the silencing of 35S rDNA loci in the S-subgenome	[165]
*Brachypodium hybridum*, *B. distachyon*, *B. stacei*	FISH with 5S and 35S rDNA-targeting probes	Comparative analysis of repetitive DNA, focusing on rDNA, in two *B. hybridum* genotypes of significantly different evolutionary ages; in the younger genotype, ABR113, partial elimination of 35S rDNA units was detected; the older genotype, Bhyb26, exhibited a tendency toward diploidisation, with a reduction in the number of both 35S and 5S rDNA loci	[166]
Multiple genotypes of *Festuca × Lolium* hybrids, *Festuca × Festuca* interspecific hybrids	FISH with a 45S rDNA-targeting probe; GISH with gDNA of *F. pratensis* and *F. glaucescens*	Investigating ND in Festulolium and fescue hybrids; providing new evidence that this phenomenon is maternity-independent, aligns with genome dominance, and occurs early after hybrid genome merging, being completed in the F_2_ generation	[167]
Two *Tragopogon porrifolius* lines, por1 and por2, which significantly differ in their 35S rDNA copy number	FISH with 5S and 35S rDNA-targeting probes	A positive correlation between the lower 35S rDNA copy number in por1 and the size of NORs on chromosomes D; both L- and S-variants of 35S rDNA were detected in por2,whereas only the S-rDNA variant was found in por1; in por1, the expression of S-rDNA was linked to secondary constrictions (SCs) of NORs located on both chromosomes A; in por2, silencing of S-rDNA was accompanied by NOR condensation on chromosomes A, the presence of SCs on D-NORs, and the expression of L-rDNA, suggesting bidirectional ND	[168]

In contrast, autotetraploids of *Saccharum spontaneum* exhibit strict control over chromosome duplication [162]. Meiotic stability was observed in a series of autopolyploid clones of *S. spontaneum*, which displayed diploid-like chromosome behaviour [67] (Figure 2K,L). Similarly, stable inheritance patterns resulting from diploidisation were detected in the majority of progeny from both synthetic and natural allotetraploid wheat hybrids [163].

The 35S rDNA sequence has been the focus of numerous studies on natural plant and resynthesised allopolyploids and hybrids, particularly regarding nucleolar dominance (ND), as recently reviewed [169]. In ND, 35S rDNA loci inherited from one progenitor are transcriptionally dominant over those from the other. For example, *B. hybridum*, a natural allopolyploid and an annual representative of the model grass genus *Brachypodium*, has long been [170] and continues to be extensively studied regarding ND, including its epigenetic landscape [41], and more recently, its tissue specificity [164], and intraspecific variation [165,166]. The contribution of parental genomes to ND has also been investigated in *Festulolium* and *Festuca* hybrids [167]. In *Tragopogon porrifolius* lines differing in their 35S rDNA composition, a positive correlation has been observed between a lower 35S rDNA copy number and the size of the nucleolus organiser region (NOR) [168]. While uniparental silencing of 35S rDNA in interspecific hybrids and allopolyploids is well-documented, evidence for similar silencing of 5S rRNA genes has long been lacking. This issue was addressed in 2024 when Mandáková et al. [171] reported the first instance of uniparental silencing of 5S rDNA in *Cardamine* polyploids, opening avenues for further research into the regulatory roles of these genes within complex polyploid genomes.

## 6. Cytogenetics-Assisted Crop Improvement

The rising demand for plant-based foods and products, set against the backdrop of climate change, presents a significant challenge for breeders, who must rapidly develop crop varieties that are more productive, resilient, and resource-efficient. Crop wild relatives (CWRs) represent a valuable reservoir of genetic diversity, particularly for improving tolerance to both biotic and abiotic stresses. In addition, they offer potential for improving yield, quality, and adaptability to harsh environmental conditions, whilst also broadening the genetic base of cultivated crops [172]. In this context, chromosome manipulation remains one of the most important tools available to plant breeders for introducing novel variation into crop varieties (for reviews, see [173,174,175]).

Genomic resources, such as genetic markers, reference genomes, transcriptomes, gene expression profiles, and protein databases, are essential tools in plant breeding. They support the identification of key traits, the analysis of genetic diversity, genomic mapping, and marker-assisted selection, and help to accelerate the development of improved cultivars. Modern genomic approaches are increasingly displacing traditional cytogenetic analyses in current research [176]. However, molecular cytogenetic studies of chromosomes in both crops and CWRs remain vital for understanding their evolution, genetic recombination patterns, and karyotypic stability [175,177]. To maintain the desirable traits bred into elite cultivars, the amount of alien genetic material introduced must be carefully regulated. For this purpose, a combination of cytomolecular tools, including FISH and GISH, is widely employed to examine and select desirable genotypes (section V of Figure 2). Examples of recent achievements in the study of breeding lines using molecular cytogenetic methods are presented in Table 5.

Cereals are the primary staple crops in most regions of the world and represent one of the crop groups in which considerable efforts have been directed towards introgression breeding. Interest in chromosome organisation in the diploid progenitors of common wheat, as well as in wild wheat species, arises largely from their value as sources of novel genes that were lost during domestication. Recent research includes the development and cytogenetic characterisation of introgression lines of bread wheat (*Triticum aestivum*) with rye [178,179,180,181], and numerous CWRs within the Triticeae tribe that exhibit various ploidy levels, such as *Agropyron cristatum* [68,182] (Figure 2M), *Aegilops biuncialis* [183], *Ae. geniculata* [69,184,185] (Figure 2N), *Elymus sibiricus* [186], *Leymus mollis* [187], *Psathyrostachys huashanica* [188,189], *Thinopyrum intermedium* [190], and *Th. ponticum* [191]. The primary traits of interest have included resistance to leaf rust, stripe rust, *Fusarium* head blight, and powdery mildew, together with desirable agronomic characteristics such as plant height, spike length, elongated glumes, and increased grain size. Similarly, chromosomal translocations carrying leaf rust resistance genes have been characterised in triticale–*Aegilops kotschyi*, and triticale–*Ae. tauschii* translocation lines [178].

**Table 5:** A selection of recent research articles on cytogenetics-assisted crop improvement.

Research Object	Research Approach	Trait(s) of Interest, Aims, and Key Findings	References
*×Triticosecale* introgression lines	GISH with gDNA of *Aegilops sharonensis* and *Ae. taushii*	Leaf rust caused by *Puccinia triticina*: identification of *Ae. kotschyi* and *Ae. tauschii* chromosome segments in triticale translocation lines carrying resistance genes	[178]
*Triticum aestivum–Secale cereale* introgression lines	GISH with gDNA of *S. cereale*; FISH with the repetitive sequence pAs1 and pSc119.2 probes; the 6c6 wheat-specific centromeric probe; the pMD-CEN3 *S. cereale*-specific centromeric probe; and an *Arabidopsis thaliana*-type (T_3_AG_3_) telomeric probe	Stripe rust caused by *Puccinia striiformis*: cytomolecular characterisation of the wheat–rye T1RS.1BL translocation line, including the presence of complex chromosome translocations	[179,180]
*×Triticosecale* × wheat derivatives	GISH with gDNA of *S. cereale*; FISH with pSc119.2, pTa71 (35S rDNA), and pAs1 probes	Yellow rust resistance: identification of the 1RS.1BL translocation in triticale × wheat progenies	[181]
*Triticum aestivum–Agropyron cristatum* introgression lines	GISH with gDNA of *A. cristatum*; oligo-FISH with pSc119.2-1, pTa531-1, pAcCR1, and CCS1 probes	Plant height and leaf size: identification of spontaneous T1AL.1PS and T1AS.1PL Robertsonian translocations in the wheat–*A. cristatum* translocation lines	[68]
*Triticum aestivum–Agropyron cristatum* introgression line	GISH with gDNA of *A. cristatum*	Multiple elite agronomic traits, including high resistance to powdery mildew and leaf rust: characterisation of the wheat–*A. cristatum* disomic 6P addition line	[182]
*Triticum aestivum–Aegilops biuncialis* introgression line	GISH with gDNA of *Ae. umbelulata*, *Ae. comosa*, and *T. turgidum*; oligo-FISH with pAs1 and pSc119.2 probes	Glume properties: characterisation of the wheat–*Ae. biuncialis* 5M^b^ disomic addition line	[183]
*Triticum aestivum–Aegilops geniculata* introgression lines	GISH with gDNA of *Ae. geniculata*; oligo-FISH with pAs1 and pSc119.2 probes	*Fusarium* head blight, powdery mildew, and stripe rust resistance: characterisation of substitution lines with high resistance to these diseases, derived from hybrid progeny between *Ae. geniculata* and hexaploid wheat	[69]
*Triticum aestivum–Aegilops geniculata* introgression lines	GISH with gDNA of *Ae. geniculata*; oligo-FISH with pSc119.2 and pTa535 probes	Stripe rust (3M^g^ DAL) and powdery mildew (7M^g^ DAL) resistance: characterisation of wheat–*Ae. geniculata* disomic addition lines	[184,185]
*Triticum aestivum–Elymus sibiricus* introgression line	GISH with gDNA of *E. sibiricus*; FISH with 35S rDNA-targeting probe	Leaf rust resistance: characterisation of the novel wheat–*E. sibiricus* 3S^t^ addition line	[186]
*Triticum aestivum–Leymus mollis* introgression line	GISH with gDNA of *L. mollis*; oligo-FISH with pSc119.2 and pTa535 probes	Stripe rust resistance, spike length: characterisation of a novel wheat–*L. mollis* 2Ns (2D) disomic substitution line	[187]
Interspecific derivatives between *Triticum aestivum* and *Psathyrostachys huashanica*	GISH with gDNA of *P. huashanica*; oligo-FISH with pSc119.2 and oligo-pTa535 probes	*Fusarium* head blight resistance: identifying and characterising two pathogen-resistant interspecific derivatives: wheat–*P. huashanica* 1Ns long arm ditelosomic addition line and 2Ns substitution line	[188]
*Triticum aestivum–Psathyrostachys huashanica* introgression line	GISH with gDNA of *P. huashanica*; oligo-FISH with pSc119.2 and pTa535 probes	Several elite agronomic traits, including elongated glumes, longer spikes, larger grains, and resistance to *Fusarium* head blight: characterisation of the wheat–*P. huashanica* 3Ns disomic 6P addition line	[189]
*Triticum aestivum × Thinopyrum intermedium* derivatives	GISH with gDNA of *Th. bessarabicum*; oligo-FISH with probes pAs1-1, pAs1-3, AFA-4, (GAA)_10_, and pSc119.2-1	*Fusarium* head blight resistance: examining the chromosome composition of five wheat–*Th. intermedium* partial amphiploids with J-genome chromosomes	[190]
*Triticum aestivum–Thinopyrum intermedium* and *Triticum aestivum–Th. ponticum* introgression lines	GISH with gDNA of *Th. bessarabicum*, *Th. intermedium*, and *Th. ponticum*; oligo-FISH with pSc119.2 and pTa535 probes	Stripe rust resistance: characterisation of wheat–*Thinopyrum* disomic substitution lines	[191]
*Brassica juncea–B. fruticulosa* introgression lines	GISH with gDNA of *B. fruticulosa* and *B. nigra*; oligo-FISH with probes designed using the *B. rapa* genome and the repetitive sequence CentBr2 probe	Mustard aphid resistance: identification of introgressions from wild species into the crop and tracking the stability of introgressed fragments of interest across generations	[192]
*Hibiscus cannabinus*	FISH with 18S-1 (35S rDNA), pXV1 (5S rDNA), and pLT11 (telomeric) probes	Initial comparative cytogenetic characterisation of kenaf landrace and breeding lines	[193]
*Phaseolus vulgaris*	FISH with 5S and 35S rDNA-targeting probes	Cytogenetic characterisation of 154 common bean accessions: high polymorphism in the number of 45S rDNA sites among the five accessions studied by FISH	[194]
*Camelina sativa*	FISH with 5S and 35S rDNA-targeting probes	Cytogenetic characterisation of nine *C. sativa* genotypes: high polymorphism in the number of 5S and 45S rDNA sites	[195]
*Veronica* species/cultivars and their progenies	FISH with 5S and 35S rDNA-targeting probes	Improving *Veronica* breeding programmes: pre-screening of hybrids, identification of true hybrids, self-pollinated progenies, and false hybrids	[196]
*Gentiana cruciata* and *G. tibetica* somatic hybrids	GISH with gDNA of *G. tibetica*; FISH with 5S and 35S rDNA-targeting probes	Cytogenetic characterisation of interspecific somatic hybrids: relatively high chromosomal stability with a predominance of *G. cruciata* chromosomes	[197]
*Lilium davidii var. unicolor*, *L. regale* and *Lilium* intersectional hybrids	GISH with gDNA of *L. longiflorum* and *L. speciosum ‘gloriosoides’*; oligo-FISH with pTA794 (5S rDNA), telomeric, and pITS probes	Improving lily breeding: characterising the genomic composition of hybrid progeny and determining the parental origin of specific chromosomes; non-denaturing FISH provides an advantage when reprobing slides	[198]
*Gossypium hirsutum–G. anomalum* chromosome segment substitution lines	Oligo-FISH with painting probes specific for chromosomes 6 (Chr. 06), 9 (Chr. 9), and 11 (Chr. 11) of *G. anomalum*	Chromosome-specific identification of *G. anomalum* introgressions in a *G. hirsutum* background, supporting the SSR and resequencing data	[199]

FISH, using various probes with particular attention to gDNA and sequences targeting 5S and 35S rDNA, has also proven useful in the characterisation of breeding lines of *Brassica juncea*–*B. fruticulosa* [192], *Hibiscus cannabinus* [193], *Phaseolus vulgaris* [194], and *Camelina sativa* [195], in supporting breeding programmes for *Veronica* species [196], and in the analysis of somatic hybrids of *Gentiana cruciata* and *G. tibetica* [197], as well as inter-sectional hybrids of *Lilium* [198]. The potential of oligo-FISH using CP probes to detect chromosome segments introgressed into *Gossypium hirsutum* from its stress-tolerant wild diploid relative *G. anomalum*, has recently been demonstrated by Xu et al. [199]. This highlighted the value of this approach as a tool to facilitate interspecific introgression breeding in this key fibre crop.

## 7. Further Current Fields of Plant Cytomolecular Research

In the previous sections, we described the latest achievements in the main research areas related to plant molecular cytogenetics. Here, we briefly highlight several additional current fields in which modern molecular cytogenetic approaches play an important role. Among these is the analysis of the repeatome, excluding studies focused on rDNA, which were discussed earlier. Repetitive DNA sequences constitute a major component of nuclear genomes, acting as a structural backbone in centromeres and telomeres, driving genome evolution, and contributing to the regulation of gene expression [200]. In some angiosperms, they may account for up to 90% of the genome [201]. Their variable abundance, high sequence diversity, and distinct chromosomal distributions contribute significantly to interspecific genome divergence and, together with polyploidy, to the remarkable range of genome sizes observed among seed plants [202]. The advent of high-throughput sequencing technologies has enabled detailed exploration of the repetitive fraction of plant genomes and facilitated comparative repeatome analyses [203]. These advances have been further supported by the development of novel computational tools, such as RepeatExplorer [204] (and its updated version, RepeatExplorer2 [205]), as well as TAREAN [206], which enable de novo identification of diverse classes of repetitive DNA elements. These tools employ sequence clustering algorithms to generate graphical representations (graph layouts) of repeat clusters based on sequence similarity and graph connectivity. Each cluster is then analysed for similarity to known repeats in existing databases, such as the database of retrotransposon protein domains (REXdb). The resulting cluster shapes are often characteristic of specific repeat types: for example, circular or ring-like graph structures typically indicate tandemly arranged satellite DNA, whereas more complex, branching patterns are commonly associated with dispersed elements, such as long terminal repeat (LTR) retrotransposons. This graphical approach thus serves as a valuable visual aid in interpreting repeatome composition and dynamics. However, the precise chromosomal localisation of predicted repeats often requires validation through FISH analysis. In recent years, the combined application of RepeatExplorer and FISH has been employed to identify and characterise DNA repeats in a range of species, including *Ensete glaucum* [207], *Juncus effusus* [208], *Cenchrus ciliaris* [209], members of the Phaseoleae tribe [210], *Cuscuta* [211], *Hydrangea* [212], *Erythrostemon hughesii* [213], and representatives of the Leptostachyus group within the genus *Phaseolus* [214]. Repetitive DNA often exhibits species-specific genomic profiles, which can aid in understanding interspecific relationships and support taxonomic classification [215]. Genome-specific repetitive sequences, when used as FISH probes, have enabled the discrimination of different genomes in polyploid *Urochloa* [216] and the tracking of intergenomic translocation patterns across various *Avena* species [217]. In *Petunia × hybrida*, the application of a set of satellite repeat families demonstrated that recent hybridisation during breeding preserved the chromosomal positions of repeats but altered their copy numbers [140]. Recent studies have also focused on the characterisation of repetitive DNA in terms of its sequence composition and its contribution to various chromosomal structures, such as centromeres. This has been extensively investigated across a range of plant species, including *Juncus effusus* [208], *Sorghum bicolor* [215], representatives of *Saccharum* [218], *Gossypium anomalum* [136], and *Populus trichocarpa* [219]. Notably, in *Triticum aestivum*, centromeric localisation of sequences other than satellite DNA has been reported, while satellite DNAs were predominantly localised to subtelomeric regions. This pattern reveals asymmetry in subtelomere organisation among the bread wheat subgenomes and suggests its potential significance in facilitating homologous chromosome recognition and pairing during meiosis [220]. Similarly, four satellite DNAs and several LTR retrotransposons have been identified in most subtelomeric regions of *Erythrostemon hughesii* chromosomes [213].

The application of molecular cytogenetic techniques is also crucial for the reliable assessment of early genetic effects on nuclear genome instability following exposure to chemical and physical mutagens, with particular emphasis on the investigation of micronuclei, as reviewed in [221]. In this context, FISH with various repetitive DNA probes has proven effective in barley [222], *Brachypodium* [223], and maize [224], with the model grass providing particularly comprehensive insight into micronuclei composition through the use of CP probes [225,226]. Furthermore, recent studies in *Brachypodium* on DNA methylation and histone modifications, using fluorescently labelled antibodies, have shed new light on the role of epigenetic regulation in micronuclei induction under mutagenic conditions [44,45]. In the future, such approaches may contribute to more accurate assessments of the impact of environmental stress on plant genome integrity.

Molecular cytogenetics provides valuable tools for dissecting various aspects of meiosis. For example, Zhang et al. [67] employed FISH with CP probes to examine chromosome behaviour during meiosis in a series of autopolyploid clones of *Saccharum spontaneum*. Despite their broad ploidy range, all clones exhibited stable, diploid-like chromosome behaviour, with homologues predominantly forming bivalents (Figure 2K). Similarly, homologous chromosome pairing has been studied in rice, where native chromosome ends have been shown to play a critical role in initiating the process [227]. Corresponding analyses in tetraploid maize highlighted the importance of DNA sequence similarity in promoting preferential homologous pairing [228]. These factors, along with other mechanisms such as the well-characterised *Ph1* locus in polyploid wheats [229] and the recently identified *BnaPh1* QTL in *Brassica napus* [230], contribute to the regulation of chromosome pairing in allopolyploids.

Recent studies have also highlighted the role of molecular cytogenetics, with particular emphasis on fluorescent immunolocalisation, in improving our understanding of various aspects of meiotic recombination in arabidopsis. For example, Blackwell et al. [231] demonstrated that the mismatch repair protein MSH2 promotes crossovers in genomic regions with higher sequence diversity. Similarly, Zhu et al. [232] reported the importance of the SMC5/6 (STRUCTURAL MAINTENANCE OF CHROMOSOMES) complex in maintaining the progression of meiotic recombination. Natural variation in the *SNI1* (*SUPPRESSOR OF NPR1-1 INDUCIBLE 1*) gene, which encodes a component of this complex, was shown to affect its function and may modulate the crossover landscape under varying environmental conditions. In their most recent work, these authors also shed light on the role of the ATR (ATAXIA TELANGIECTASIA AND RAD3-RELATED) kinase, whose inactivation leads to a marked redistribution of crossovers, with a decrease in pericentromeric regions and an increase within the chromosome arms [233].

## 8. Concluding Remarks and Future Perspectives

Over the past decade, plant molecular cytogenetics has undoubtedly undergone significant actual or potential breakthroughs, driven by advances in genomic and molecular methodologies. One such breakthrough was the introduction and widespread adoption of oligo-FISH-based CP, which enabled the expansion of complex nuclear genome analyses at the microscopic level beyond a limited number of small-genome models, such as arabidopsis and *Brachypodium* and their relatives, to dozens, if not hundreds, of species, including both non-model and crop species representing a wide range of angiosperm families and nuclear genome sizes. On the other hand, some initially promising approaches, such as CRISPR/Cas [234,235]-based live-cell imaging, have not fulfilled all expectations. This technique utilises a fluorescence-tagged, nuclease-deficient Cas (dCas) protein to track specific DNA sequences in vivo in a programmable manner, and it holds considerable potential for visualising various aspects of nuclear dynamics (for review, see [236]). Although this approach has proven successful in human [237] and murine [238] living cells, its application in plants has so far been limited to the demonstration of telomere dynamics in *Nicotiana benthamiana* [239]. A similar attempt in arabidopsis [240] and brachypodium resulted in ectopic distribution of GFP signals, exposing the limitations of this methodology in small-genome plants, although it may retain some potential when applied to fixed material [241].

The CRISPR/Cas system has also recently been applied to chromosome-scale engineering through the generation of targeted CRs [242]. As demonstrated in arabidopsis, inversions induced using this approach can influence local recombination patterns, either restoring meiotic crossovers in chromosomal regions that were previously inert to genetic exchange [243], or, conversely, massively suppressing meiotic recombination [244]. This new method of manipulating chromosomes holds considerable potential in plant biology and biotechnology. Its applications include breaking genetic linkages between specific genes, reversing natural inversions that suppress recombination in breeding programmes, artificially establishing genetic isolation, and constructing mini-cargo chromosomes, as reviewed by Puchta and Houben [245]. The most recent studies highlight the usefulness of targeted chromosome engineering for investigating telomere dynamics, chromatin structure, gene expression, and phenotypic stability in arabidopsis [246], and are already providing valuable materials and inspiration for advanced cytomolecular analyses [247].

Another potentially significant breakthrough may lie in the application of AI to cytogenetics, particularly in the context of advanced image analysis [248]. One especially promising development is the integration of deep learning-based AI algorithms into karyotyping software. As recently reviewed by Rosenblum et al. [249], four commercially available AI-assisted karyotyping platforms are already in use for the analysis of human chromosomes in both clinical and research contexts. These systems address various limitations inherent in traditional automated karyotyping, particularly those related to image acquisition, segmentation, chromosome classification, and analytical accuracy, and they hold the potential to redefine the current paradigm of chromosome analysis. Similar approaches could also hold transformative potential in plant systems, although their implementation is likely to be considerably more challenging due to the vast number of plant species that may serve as targets for analysis, as well as the extensive diversity of their karyotypes. Nevertheless, an emerging AI-based image analysis pipeline is currently under development as part of the fifth generation of the long-established CHIAS chromosome image analysis system [250,251,252,253], and it has already yielded encouraging preliminary results for potential application in plant cytogenetics. Furthermore, Oxford Instruments reports the integration of AI into the latest release of its Imaris imaging package [51]. Thus, in light of the current body of evidence, the future of cytomolecular analyses in plants appears prospective, particularly as an effective means of bridging genomic and transcriptomic data with advanced microscopy-based observations and interpretations.

## Abbreviations

The following abbreviations are used in this manuscript:

2D	Two-dimensional
3D	Three-dimensional
3C	Chromosome conformation capture
ACK	Ancestral Crucifer Karyotype
AI	Artificial intelligence
APK	Ancestral Phaseoleae Karyotype
arabidopsis	*Arabidopsis thaliana*
BAC	Bacterial artificial chromosome
*Brachypodium*	*Brachypodium distachyon*
Cas	CRISPR-associated protein
CCP	Comparative chromosome painting
CENH3	Centromeric histone H3 variant
ChIA-PET	Chromatin interaction analysis by paired-end tag sequencing
CP	Chromosome painting
CR	Chromosome rearrangement
CRISPR	Clustered regularly interspaced short palindromic repeats
CT	Chromosome territory
CWR	Crop wild relative
dCas	Nuclease-deficient (‘dead’) CRISPR-associated protein
EdU	5-ethynyl-2′-deoxyuridine
FISH	Fluorescence in situ hybridisation
FITC	Fluorescein isothiocyanate
gDNA	Total genomic DNA
GISH	Genomic in situ hybridisation
Hi-C	High-throughput chromatin conformation capture
HiChIP	In situ Hi-C followed by chromatin immunoprecipitation
HVTEM	High-voltage transmission electron microscopy
ISH	In situ hybridisation
LTR	Long terminal repeat
MY	Million years
ND	Nucleolar dominance
Oligo-FISH	Oligonucleotide fluorescence in situ hybridisation
rDNA	Ribosomal DNA
REXdb	Database of retrotransposon protein domains
rRNA	Ribosomal RNA
SEM	Scanning electron microscopy
SIM	Structured illumination microscopy
TEM	Transmission electron microscopy
Topo	Topoisomerase
UHVTEM	Ultra-high-voltage transmission electron microscopy
WT	Wild type
